# Upper Extremity Motor Evoked Potentials and Hand Function in Elderly Stroke Survivors: A Correlational Study

**DOI:** 10.3390/jcm15041467

**Published:** 2026-02-13

**Authors:** Woo-Hwa Choi, Jae-Eun Park, Seong Jin, Hyun-Ah Lee, Jong-Hu Jeon, Byeong-Wook Lee, Ji-Yeon Oh, Eui-Jin An, Ho-Yong Jeong, Ji-Su Choi, Young Lee

**Affiliations:** 1 Department of Physical Medicine and Rehabilitation, Veterans Health Service Medical Center, Seoul 05368, Republic of Korea; cwh5023@gmail.com (W.-H.C.);; 2 Veterans Medical Research Institute, Veterans Health Service Medical Center, Seoul 05368, Republic of Korea

**Keywords:** stroke rehabilitation, motor evoked potentials, upper extremity function, hand function

## Abstract

**Background/Objectives:** The impact of stroke on upper extremity function in the older adult population underscores the need for accurate recovery prediction. Motor evoked potential (MEP) has been explored as a predictor of upper extremity function recovery in patients with stroke. However, research specifically targeting the geriatric population remains limited. Therefore, this study focused specifically on patients aged 65 years and older to investigate correlations between MEP parameters and upper extremity function. This study investigates correlations between MEP parameters (amplitude and latency) and upper extremity function-related measures, including Medical Research Council (MRC) scale, the Korean version of the Modified Barthel Index (K-MBI), and the Hand Function Test (HFT), including grip strength, pinch strength, the Box and Block Test, and the 9-Hole Peg Test, in older adults with stroke. **Methods:** A multiple linear regression model predicts upper extremity outcomes using initial MEP parameters, time, and function. The dataset includes 90 patients with stroke categorized by timing of the first MEP assessment: ≤3 months (*n* = 42) or >3 months (*n* = 48). **Results:** MEP amplitude and latency were significantly correlated with upper extremity function in both groups. No significant correlations were found between MEP amplitude or latency and outcome measures. Regression analysis showed that initial MEP amplitude had a limited association with outcomes, whereas latency was significantly associated with grip strength (β: −10.205, 95% CI: −19.374~−1.036) and the Box and Block Test (β: −10.204, 95% CI: −20.254~−0.154). Initial upper extremity parameters were significantly associated with K-MBI and HFT follow-up results (*p* < 0.05). **Conclusions:** Larger MEP amplitude and faster initial MEP latency were associated with improved upper extremity function in patients with stroke. In older patients, MEP latency, rather than amplitude, demonstrated greater predictive value for upper extremity function recovery, possibly due to age-related muscle atrophy, a factor not fully addressed in existing prognostic frameworks such as PREP2. These findings support the integration of MEP latency assessment into geriatric stroke prognostication, complementing existing frameworks such as PREP2, and may guide personalized rehabilitation planning to optimize functional recovery and independence.

## 1. Introduction

Stroke remains a leading cause of severe long-term disability, with its global incidence and socioeconomic burden continuing to rise alongside the aging population [1,2]. A critical challenge in post-stroke rehabilitation is the accurate prediction of motor recovery, particularly for the upper extremities, which are essential for independence in activities of daily living [3,4]. While approximately 50–80% of patients experience upper extremity impairment in the acute phase [5,6], recovery in this area is often slower and less predictable than in the lower limbs [7,8].

Neurophysiological techniques, specifically Motor Evoked Potentials (MEPs) induced by Transcranial Magnetic Stimulation (TMS), have emerged as powerful tools for assessing the functional integrity of the corticospinal tract (CST) [9,10]. The primary strength of MEP testing lies in its ability to provide an objective, quantitative measure of motor pathway connectivity [11]. It has been established that the presence of an MEP in the early stages of stroke is a strong positive prognostic indicator for motor recovery [12,13,14], often demonstrating greater predictive value than clinical scales or other neurophysiological tests like somatosensory-evoked potentials (SSEPs) [15].

Despite these strengths, several open questions remain regarding the optimal use of MEPs in stroke prognosis. While the presence or absence of an MEP is widely utilized [16,17,18,19], modern predictive frameworks, such as the PREP2 algorithm, emphasize the presence of MEPs as a primary biomarker for recovery potential [20]. However, these models have not fully accounted for the physiological changes associated with aging, such as muscle atrophy and age-dependent increases in motor thresholds [21,22]. Whether specific MEP characteristics like latency or amplitude serve as more reliable predictors in the geriatric population remains a critical gap in current research [23,24]. Therefore, this study aimed to investigate the correlation between MEP parameters and upper extremity function, specifically in stroke patients aged 65 years and older.

## 2. Materials and Methods

### 2.1. Study Design

Assessment of upper extremity function involved the Medical Research Council (MRC) scale, the Korean version of the Modified Barthel Index (K-MBI), and the Hand Function Test (HFT), which includes grip strength, pinch strength, the Box and Block Test, and the Nine-hole Peg Test. The patients were categorized into two distinct groups based on the duration between stroke onset and the initial MEP measurement.

Notably, findings from the Copenhagen Stroke Study [25] indicate that approximately 95% of stroke survivors attain their optimal neurological status within 11 weeks of stroke onset. Concurrently, other research highlights that a substantial portion of upper extremity motor recovery occurs within the initial 4 weeks and stabilizes around the 3-month mark poststroke [26]. Eighty percent of patients achieved maximum upper extremity recovery during the 3rd week, with this proportion increasing to 95% by the 9th week [27].

Based on this understanding of motor recovery, we divided our patients into two groups based on whether their first MEP measurement occurred within 3 months of stroke onset. This decision was made because most motor recovery occurs within this time frame, with minimal subsequent changes in upper extremity function outcomes expected [25,26,27].

### 2.2. Study Participants

**Figure 1:**
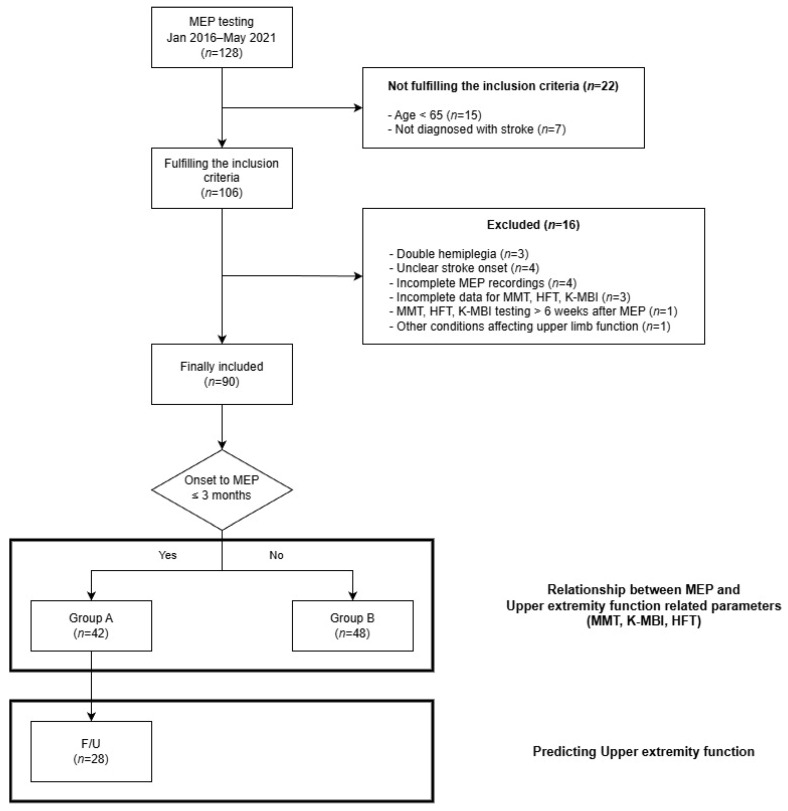
Flowchart for patient inclusion, exclusion and study method. Abbreviations: MEP, Motor Evoked Potential; HFT, Hand Function Test; MMT, Manual Muscle Test; K-MBI, Korean version of the Modified Barthel Index; F/U, Follow Up.

Between January 2016 and May 2021, a total of 128 patients who had undergone MEP testing at the Veterans Health Service Medical Center were retrospectively identified. Patients were included in the study if they met the following criteria: (1) aged 65 years or older, and (2) a diagnosis of stroke. Among the 106 patients who met the inclusion criteria, some were excluded based on the following exclusion criteria: (1) presence of other conditions that could affect upper limb function (1 patient); (2) double hemiplegia (3 patients); (3) unclear stroke onset time (4 patients); (4) incomplete or uncooperative MEP recordings (4 patients); (5) incomplete or missing data for manual muscle test (MMT), Hand function tests, or K-MBI (3 patients); (6) MMT, hand function tests, or K-MBI performed more than 6 weeks after MEP testing (1 patient). Ultimately, 90 patients were included in this study (Figure 1).

### 2.3. Clinical Assessment

The MEP is a neurophysiological test that evaluates motor pathway function by measuring the muscle response to electrical stimulation. Surface electrodes were placed on the abductor digiti minimi (ADM) muscle of each hand, and magnetic stimulation was delivered over the bilateral primary motor cortex and Erb’s point using a Magstim Rapid stimulator (Magstim Company Ltd., Whitland, UK).

In this study, the ADM was specifically selected as the target muscle to record MEP parameters. While the abductor pollicis brevis (APB) is commonly utilized in the literature, the ADM provides superior signal stability in geriatric populations and minimizes confounding effects from peripheral pathologies, such as carpal tunnel syndrome, which are highly prevalent in older adults [28]. The MEP measurements were obtained by stimulating the primary motor cortex with seven stimuli at 120% of the lowest resting motor threshold (RMT). Notably, the RMT was determined independently for each hemisphere (unaffected and affected) to account for inherent differences in cortical excitability between the two sides. To ensure the comparability of MEP parameters, the stimulator output intensity required to reach the threshold was documented and incorporated as a covariate in the statistical models. The mean amplitudes and latencies of five responses were calculated after excluding extreme characteristics to ensure consistency [29].

This study also assessed the M-wave, a neurophysiological parameter representing the electrical response of peripheral nerves to muscles, reflecting overall muscle activity and baseline electrophysiological properties. The M-wave amplitude was obtained by delivering three stimuli over Erb’s point and was compared with responses from primary motor cortex stimulation to calculate the A/M ratio. This ratio indicates the connectivity between the central nervous system and muscles and provides insights into synaptic connectivity and motor pathway changes.

Evoked potentials were recorded by an experienced rehabilitation physician using the CareFusion Nicolet EDX system with Viking Software (Version 20.1.30) to ensure accuracy. The study included 90 patients with stroke who were divided into groups based on stroke onset time, thereby providing comprehensive data on motor pathway function.

MMT is an assessment used to measure the voluntary contraction ability of an individual muscle or a group of muscles acting on a particular joint. Muscle strength was evaluated using the Medical Research Council Scale (MRC) for the shoulder, elbow, wrist, and fingers of the upper limbs, as well as the hip, knee, ankle, and toes of the lower limbs. Each muscle group was scored on a scale ranging from 0 to 5 points, reflecting different levels of muscle strength. For both the upper extremity (MMT UE) and lower extremity (MMT LE), muscle strength scores were averaged and categorized into six groups: Z = 0, T = 1, P = 2, F = 3, G = 4, and N = 5.

The Fugl–Meyer test is commonly used to evaluate hand function; however, the results may be affected by the examiner’s subjective judgment. Therefore, four objective measures—grip strength, pinch strength, the Box and Block Test, and the 9-Hole Peg Test—were used to assess finger muscle strength and dexterity [30].

Grip strength was measured in pounds (lbs) by averaging three measurements obtained using a handheld dynamometer. Pinch strength was measured in lbs by averaging the three lateral pinch measurements obtained using a pinch gauge. The Box and Block Test evaluates upper extremity dexterity by counting the number of 1-inch blocks transferred within 1 min. The 9-Hole Peg Test assesses finger dexterity by measuring the time (in 10-ms units) required to pick up and insert pegs into holes on a wooden board.

In cases where the 9-Hole Peg Test was not performed, the performance time was recorded as 0 ms. We divided the results into five categories using quartiles and performed a statistical analysis accordingly to address this issue. Grip strength, pinch strength, and Box and Block Test results were analyzed as continuous variables.

The Modified Barthel Index (MBI) assesses the activities of daily living with a maximum score of 100 points. Scores were categorized into five groups based on the level of assistance required [31]. In this study, the scores were used as continuous variables for detailed analysis. As this study targeted Korean individuals, we used the Korean version of the Modified Barthel Index (K-MBI), which has been standardized in Korean, with questions that align with Korean culture and lifestyle [32].

### 2.4. Statistical Analysis

For comparison between the unaffected and affected sides, we conducted normality tests using the Shapiro–Wilk test. Paired *t*-tests were used for normally distributed data, and Wilcoxon signed-rank tests were used for non-normally distributed data. For categorical variables, such as sex, hemiplegic side, and MMT categories, we used the chi-square test or Fisher’s exact test. For continuous variables, including MEP amplitude, latency, M wave, K-MBI, and HFT (grip strength, pinch strength, Box and Block Test, and 9-Hole Peg Test), normality was assessed using the Shapiro–Wilk test. Two-sample *t*-tests or Mann–Whitney U tests were used to calculate *p*-values. We calculated correlation using Spearman correlation for between MEP and MMT, K-MBI, and HFT, and Fisher, R.A. (1992) [33] method was used to compare correlations between groups. We used multiple linear regression to investigate the association between the initial MEP and follow-up K-MBI and HFT. All analyses were performed using R software version 4.1.2. (R Foundation for Statistical Computing, Vienna, Austria). The significance level was set at *p* < 0.05.

To predict follow-up functional outcomes, we developed multiple linear regression models using initial MEP parameters. The follow-up functional outcome ($y_{2}$) was predicted by incorporating the initial MEP parameter (x), the interval between stroke onset and the MEP test (t), and the initial functional score ($y_{1}$). The regression model was defined as follows:

$$y_{2}=\beta_{0}+\;\beta_{1}x\;+\;\beta_{2}t\;+\;\beta_{3}y_{1}\;+\;\varepsilon$$

Two separate models were constructed: one based on MEP amplitude as a continuous variable, and another based on MEP latency. To account for ‘no response’ cases in the latency analysis, participants were categorized based on the median latency value, including a distinct category for those with no response. Furthermore, to control for individual differences in cortical excitability, the stimulator output intensity used during the test was included as a covariate in the amplitude model.

Sample Size Considerations: As this was a retrospective study, an a priori sample size calculation was not performed. To assess the magnitude and precision of our findings, we report 95% Confidence Intervals (CIs) for all regression coefficients in the subsequent results section rather than post-hoc power calculations.

Handling of 9-Hole Peg Test Data: For patients unable to perform the 9-Hole Peg Test, we coded these cases as Category 5 (worst performance) for correlation analyses to capture the full severity spectrum. However, in linear regression models, these cases were treated as missing data to preserve model validity and avoid violations of linearity assumptions.

## 3. Results

We measured the electrophysiological parameters of the affected and unaffected sides of older adults with stroke (Table 1). We used the median [25th–75th percentile] (minimum–maximum) to describe the data and excluded cases with ‘no response’ latency values from the analysis. We found significant differences in the MEP latency (*p* = 0.002), MEP amplitude (*p* < 0.001), and MEP amplitude/M-wave amplitude ratio (*p* < 0.001) between the two sides. However, no significant difference was observed in M-wave amplitude between the two sides (*p* = 0.242).

For the first analysis, we divided patients with stroke into two groups based on the time from stroke onset to the first MEP: Group A (≤3 months, 42 patients) and Group B (>3 months, 48 patients), and MEP measurements and upper extremity functional parameters were compared between the groups (Table 2).

The value was calculated after excluding cases where the latency was no response or the 9-Hole Peg Test was 0 (recorded as 0 if the test was not performed) to represent the median [25th–75th percentile] (minimum–maximum).

The results showed a significant difference between the groups in the time from stroke onset to the first MEP (*p* < 0.001), as presented in Table 2. However, there were no significant differences in age, sex, hemiplegic side, MEP amplitude, or MEP latency between the two groups. In addition, no significant differences were observed in upper-extremity functional parameters, including MMT, K-MBI, and HFT.

Next, we examined how MEP parameters (amplitude and latency) were related to upper extremity function test results (MMT, K-MBI, and HFT) in patients with stroke by comparing the two groups based on how long it took them to obtain their first MEP after stroke onset (Table 3).

For the analysis, we divided the results into five groups based on the quartiles of the 9-Hole Peg Test and latency. The 9-Hole Peg Test scores were coded from 1 to 4 based on the quartile ranking, and a code of 5 was assigned when the test was not conducted (recorded as 0). Latency parameters classified as ‘no response’ were coded in the same manner, with a value of 5 assigned to these cases.

In Group A (≤3 months, 42 patients), we found significant positive correlations between amplitude and upper/lower MMT (r = 0.62 and r = 0.39, both *p* < 0.05). Positive correlations were also observed between MEP amplitude and HFT measures, including grip strength, pinch strength, and Box and Block Test (r = 0.69, r = 0.72, and r = 0.61, respectively; all *p* < 0.05). However, MEP amplitude showed a significant negative correlation with the 9-Hole Peg Test (r = −0.64, *p* < 0.05), indicating that higher test performance was associated with lower amplitude parameters.

Latency was significantly negatively correlated with upper and lower MMT, K-MBI, and HFT measures, including grip strength, pinch strength, and Box and Block Test (r = −0.58, r = −0.34, r = −0.47, r = −0.62, r = −0.64, and r = −0.5, respectively; all *p* < 0.05). However, latency showed a significant positive correlation with the 9-Hole Peg Test (r = 0.49, *p* < 0.05), suggesting that shorter latency was associated with better test performance.

In Group B (>3 months, 48 patients), there were significant positive correlations between amplitude and upper/lower MMT (r = 0.72 and r = 0.58, both *p* < 0.05), as well as with various HFT measures such as grip strength, pinch strength, and box and block (r = 0.7, r = 0.69, and r = 0.62, all *p* < 0.05). However, MEP amplitude was significantly negatively correlated with the 9-Hole Peg Test (r = −0.59, *p* < 0.05).

Similarly, latency significantly negatively correlated with upper and lower MMT and HFT measures, including grip strength, pinch strength, and the Box and Block Test (r = −0.7, r = −0.57, r = −0.66, r = −0.63, and r = −0.59, respectively; all *p* < 0.05). However, latency showed a significant positive correlation with the 9-Hole Peg Test (r = 0.54, *p* < 0.05). Additionally, in Group B, K-MBI did not significantly correlate with either MEP amplitude (r = 0.25, *p* > 0.05) or latency (r = −0.26, *p* > 0.05).

There were no significant correlations between the amplitudes or latencies of any measured variables when the two groups were compared.

For the second study, we selected 42 patients who underwent their first MEP test within 3 months of stroke onset (Group A). Among these patients, 28 underwent follow-up assessments. If a patient underwent three or more follow-up visits during the study period, the visit with the fewest missing data points was selected. In cases in which the amount of missing data was equal across visits, the earlier visit was selected.

We conducted a multiple linear regression analysis to examine whether favorable initial amplitude or latency parameters were associated with clinically meaningful improvements in subsequent K-MBI and HFT outcomes. Latency parameters, including those recorded as ‘no response’, were divided into three categories based on the median (coded as x_1_ for values below the median, x_2_ for values above the median, and x_3_ for latency recorded as ‘no response’). Unlike the correlation analysis, patients who did not complete the 9-Hole Peg Test were treated as having missing data, and the outcome data were transformed using a logarithmic function. The MMT was excluded from the analysis because certain score values occurred infrequently, resulting in sparse data.

The multiple linear regression analysis was performed to identify whether initial MEP characteristics could independently predict follow-up outcomes. In the amplitude-based model, initial amplitude parameters did not show a statistically significant association with follow-up K-MBI or HFT outcomes (*p* > 0.05), after adjusting for test timing and baseline functional levels (Table 4). In contrast, the latency-based model revealed that initial MEP characteristics were significant predictors for specific hand functions. Specifically, longer latency or the absence of a response was negatively associated with follow-up grip strength (β: −10.205, *p* = 0.031) and Box and Block Test scores (β: −10.204, *p* = 0.047) (Table 5). These results indicate that initial latency parameters provide higher prognostic value for manual dexterity than amplitude in the older adult population.

## 4. Discussion

Given the aging population and the increasing number of stroke survivors, predicting the degree of future motor function recovery in older adults with stroke is essential for establishing overall treatment goals and individualized care plans. It is well established that the severity of early upper limb functional impairment is a strong predictor of recovery [34], and previous research has suggested that MEP testing at an early stage can also predict motor function recovery [35]. However, few studies have specifically examined older adults with stroke. Our study aimed to build upon this existing evidence by providing further insights into the predictive value of MEP testing in older adults with stroke, specifically those aged 65 years or older.

### 4.1. Relationship Between MEP and Upper Extremity Function

We observed significant correlations between MMT scores and MEP parameters in both the upper and lower limbs across both groups. However, the correlations were notably stronger for the upper limb than for the lower limb. Given that MEPs were recorded from the abductor digiti minimi (ADM) muscle, a stronger association with upper extremity function—particularly hand dexterity—is physiologically expected.

Nevertheless, the correlation with lower-limb MMT scores suggests that the MEP characteristics of the upper limb may serve as a proxy for the overall functional integrity of the corticospinal tract (CST). Previous studies have demonstrated that MEPs obtained from the upper extremity can indeed reflect general motor pathway recovery and are associated with lower-limb motor scores in stroke patients. Specifically, Kim et al. reported that upper-limb MEP responses in the acute stage were significantly correlated with both upper- and lower-extremity motor functions [16]. This systemic correlation likely reflects the extent of the primary brain lesion affecting the broad distribution of the CST.

### 4.2. Integration with the PREP2 Algorithm and Prognostic Value

A critical question is how our findings align with established prognostic frameworks, such as the PREP2 algorithm [20]. The PREP2 model utilizes the presence or absence of an MEP as a primary biomarker for recovery potential. While our data support the fundamental premise of PREP2—that CST integrity is a primary biomarker—our results suggest that in the geriatric population, the specific parameter of latency may offer more nuanced predictive value than amplitude alone.

In older adults, initial MEP latency demonstrated a statistically significant negative association with follow-up hand function characteristics [24]. The superiority of latency as a prognostic marker in older adults can be attributed to several age-related physiological factors:

Muscle Fiber Atrophy: Aging leads to a progressive reduction in the number and size of type 2 (fast-twitch) muscle fibers [36,37]. This neurogenic process results in a significantly lower baseline for MEP amplitude characteristics, which may obscure the true state of corticospinal tract (CST) integrity in older survivors.

Stability of Central Conduction: While muscle mass and fiber excitability (which determine amplitude) fluctuate significantly with age and stroke-related atrophy, the myelination and axonal diameter of the remaining CST fibers (which determine latency) remain relatively more stable indicators of motor pathway continuity [38].

Central vs. Peripheral Contribution: In the elderly, a reduced amplitude may reflect peripheral muscle sarcopenia rather than central damage. Conversely, prolonged latency or a ‘no response’ state more accurately represents a failure in central motor signal conduction through the CST.

By focusing on latency, clinicians can bypass the ‘floor effect’ often seen in amplitude measurements of atrophied muscles, thereby achieving a more reliable forecast of the patient’s capacity for motor learning and daily autonomy [39,40].

### 4.3. Clinical Implications: Motor Learning and Daily Autonomy

The neurophysiological evidence presented here has profound implications for motor learning and the restoration of daily autonomy. The integrity of the motor pathway, as evidenced by favorable MEP parameters, is a prerequisite for the plastic changes associated with repetitive task-specific training [41]. Patients with preserved latency characteristics likely possess the neural substrate required for effective motor learning during rehabilitation.

Furthermore, beyond pure motor conduction, clinical recovery is a multidimensional process influenced by cortical plasticity and psychological factors [39], and non-motor sequelae such as post-stroke fatigue. Recent studies highlight that fatigue is highly prevalent in older survivors and strongly associated with perceived recovery [42]. Therefore, neurophysiological markers should ideally be integrated with assessments of fatigue, sleep quality, and emotional well-being to inform more holistic, fatigue-sensitive rehabilitation planning [40,42].

### 4.4. Limitations

Several limitations of this study should be acknowledged. First, the small sample size and single-center design may limit the generalizability of our findings to broader populations or different healthcare settings. Further research with larger, multi-center cohorts is needed to confirm the utility of latency as a parameter for assessing upper extremity function in older stroke patients.

Second, the retrospective nature of the study inherently restricted the availability of certain clinical variables. Some assessment scales were not consistently administered, and detailed stroke lesion mapping, stroke subtype (ischemic vs. hemorrhagic), and quantitative rehabilitation intensity were not uniformly available, limiting our ability to correlate MEP findings with specific anatomical damage patterns. Furthermore, we did not account for potential confounding factors such as cognitive function or depression, which may influence functional recovery.

Third, there were methodological constraints regarding the assessment tools. While the K-MBI provides a general overview of daily activities, its broad scope makes it challenging to isolate specific upper limb functions. Additionally, MEP recordings were limited to the abductor digiti minimi (ADM) muscle; future studies should consider multi-muscle recordings for a more comprehensive assessment of corticospinal tract integrity.

Furthermore, the regression analysis was restricted to the subacute group (≤3 months, *n* = 28 with follow-up data), where ongoing recovery is most clinically relevant. This limited sample size reduces statistical power and may affect the generalizability of predictive findings.

## 5. Conclusions

With the rapid aging of the population and the subsequent rise in stroke survivors, the ability to accurately predict motor recovery is essential for establishing effective, personalized rehabilitation goals. This study demonstrates that in older stroke patients, larger MEP amplitudes and shorter latencies are significantly associated with superior upper extremity function, regardless of the time since stroke onset. Notably, MEP latency emerged as a meaningful prognostic predictor of functional improvement. A key strength of this research lies in its methodological approach, analyzing MEP parameters as both continuous and categorical variables. This dual analysis provides a more nuanced understanding of recovery prospects and enables more precise prognostication of hand function outcomes. Furthermore, our findings complement and extend existing prognostic models, such as PREP2, by highlighting that in geriatric populations, latency parameters may offer superior predictive value over amplitude alone, as they better account for age-related physiological changes. Despite the inherent limitations of a retrospective, single-center design, these insights underscore the clinical utility of MEP testing—particularly latency—as a valuable noninvasive tool. Incorporating these parameters into routine assessments will empower clinicians to formulate more comprehensive treatment plans and targeted interventions, ultimately improving the quality of life for older adult stroke survivors. Future prospective studies are warranted to further validate these findings across more diverse clinical settings.

## Figures and Tables

**Table 1:** Electrophysiological parameters in the affected and unaffected sides of older adults with stroke.

	Unaffected Side	Affected Side	*p*-Value
MEP (Latency, ms)	24.45 [22.76–25.90] (19.50–37.30)	24.60 [23.45–27.57] (21.30–39.13)	0.002 *
MEP (Amplitude, mV)	0.38 [0.23–0.72] (0.00–3.00)	0.10 [0.00–0.32] (0.00–4.30)	<0.001 *
M wave (Amplitude, mV)	4.96 [2.92–7.67] (0.50–36.00)	4.91 [2.52–7.64] (0.50–11.17)	0.242
MEP (Amp)/M wave (Amp)	0.09 [0.04–0.24] (0.00–1.09)	0.02 [0.00–0.09] (0.00–1.41)	<0.001 *

Values are presented as Median [25-percentile–75-percentile] (min–max), MEP = motor-evoked potential, * mean *p* < 0.05.

**Table 2:** Baseline characteristics, results of MEP measurements, and upper extremity function-related parameters in patients with stroke with time of stroke onset less than 3 months (Group A) and more than 3 months (Group B).

	Group A (*n* = 42)	Group B (*n* = 48)	*p*-Value
Age (year)	72.96 [69.74–75.78] (67.12–88.08)	71.51 [69.35–73.86] (65.47–88.09)	0.141
Onset to first MEP (month)	1.08 [0.84–1.20] (0.36–2.64)	13.80 [6.84–44.64] (3.00–336.00)	<0.001 *
Sex			0.661
Male	39 (92.86%)	46 (95.83%)	
Hemiplegic side			0.703
Right	21 (50.00%)	27 (56.25%)	
MEP			
Amplitude (mV)	0.10 [0.00–0.24] (0.00–1.23)	0.03 [0.00–0.43] (0.00–4.30)	0.943
Latency (ms)	24.70 [24.02–27.37] (21.30–35.00)	24.40 [22.90–28.40] (21.40–39.13)	0.970
MMT (Average)			
Upper (Affected side)	3.00 [2.00–4.00] (0.00–5.00), 2.762 ± 1.462	3.00 [1.00–4.00] (0.00–5.00), 2.708 ± 1.352	0.839
Lower (Affected side)	3.00 [2.00–4.00] (0.00–5.00), 2.881 ± 1.383	3.00 [2.00–4.00] (1.00–5.00), 2.938 ± 1.060	0.990
K-MBI	54.00 [31.50–67.00] (3.00–92.00)	61.00 [45.25–79.75] (4.00–97.00)	0.096
HFT			
Grip strength (lbs.)	13.33 [0.00–41.67] (0.00–81.67)	1.50 [0.00–41.25] (0.00–81.33)	0.517
Pinch strength (lbs.)	4.00 [0.00–12.00] (0.00–21.33)	2.17 [0.00–10.25] (0.00–20.00)	0.445
Box and block (n)	16.00 [0.00–28.50] (0.00–56.00)	0.00 [0.00–26.75] (0.00–53.00)	0.313
9-hole pegboard (ms)	4775.00 [3665.50–7168.00] (2094.00–24,222.00)	4659.00 [3544.00–5150.00] (2100.00–9875.00)	0.319

Values are presented as Median [25 percentile–75 percentile] (min–max). In order to better show the characteristics of the variables, the mean ± SD was additionally marked for MMT. MEP, motor-evoked potential; MMT, manual muscle test; K-MBI, Korean version of the Modified Barthel Index; HFT, Hand Function Test; * *p* < 0.05.

**Table 3:** Correlation between MEP (Amplitude, Latency) and upper extremity function-related parameters (MMT, K-MBI, HFT) in patients with stroke.

	Amplitude			Latency		
	Group A	Group B	*p*-Value	Group A	Group B	*p*-Value
MMT (affected upper)	0.62 *	0.72 *	0.397	−0.58 *	−0.7 *	0.347
MMT (affected lower)	0.39 *	0.58 *	0.231	−0.34 *	−0.57 *	0.170
K-MBI	0.4 *	0.25	0.440	−0.47 *	−0.26	0.273
HFT						
Grip strength (lbs.)	0.69 *	0.7 *	0.952	−0.62 *	−0.66 *	0.790
Pinch strength (lbs.)	0.72 *	0.69 *	0.786	−0.64 *	−0.63 *	0.975
Box and block (n)	0.61 *	0.62 *	0.979	−0.5 *	−0.59 *	0.553
9-hole pegboard (ms)	−0.64 *	−0.59 *	0.698	0.49 *	0.54 *	0.762

Group A, Onset to first MEP ≤ 3 months (*n* = 42); Group B, Onset to first MEP > 3 months (*n* = 48); *p*-value, Difference by chance between two groups; MMT, Manual Muscle Test; K-MBI, Korean version of Modified Barthel Index; HFT, Hand Function Test; * mean *p* < 0.05.

**Table 4:** Relationship between initial MEP amplitude and follow-up outcomes on K-MBI and HFT in follow-up patients.

	Beta (β)	95% CI (Lower, Upper)	*p*-Value
K-MBI			
Initial Amplitude (mV)	5.950	(−10.076, 21.976)	0.451
Initial Time (day)	0.143	(−0.219, 0.504)	0.423
Initial K-MBI	0.903	(0.693, 1.112)	<0.001 *
Grip strength			
Initial Amplitude (mV)	10.066	(−10.113, 30.244)	0.310
Initial Time (day)	−0.067	(−0.394, 0.260)	0.673
Initial grip strength (lbs.)	0.815	(0.587, 1.043)	<0.001 *
Pinch strength			
Initial Amplitude (mV)	1.516	(−7.190, 10.221)	0.720
Initial Time (day)	−0.043	(−0.176, 0.090)	0.510
Initial pinch strength (lbs.)	0.802	(0.459, 1.146)	<0.001 *
Box and block			
Initial Amplitude (mV)	11.425	(−7.008, 29.858)	0.210
Initial Time (day)	−0.102	(−0.429, 0.225)	0.521
Initial box and block (n)	0.786	(0.504, 1.069)	<0.001 *
9-hole pegboard			
Initial Amplitude (mV)	−0.016	(−0.607, 0.576)	0.954
Initial Time (day)	−0.002	(−0.016, 0.012)	0.721
Initial 9 peg (log_ms)	0.500	(0.203, 0.797)	0.004 *

K-MBI, Korean version of Modified Barthel Index; β, Regression coefficient; 95% CI, The lower and upper bounds of the confidence interval; * mean *p* < 0.05.

**Table 5:** Relationship between initial MEP latency and follow-up outcomes on K-MBI and HFT in follow-up patients.

	Beta (β)	95% CI (Lower, Upper)	*p*-Value
K-MBI			
Initial Latency x_1_	(Ref)		
Initial Latency x_2_ (ms)	6.184	(−4.819, 17.188)	0.257
Initial Latency x_3_ (ms)	−6.795	(−17.315, 3.726)	0.195
Initial Time (day)	0.095	(−0.248, 0.437)	0.573
Initial K-MBI	0.859	(0.655, 1.062)	<0.001 *
Grip strength			
Initial Latency x_1_	(Ref)		
Initial Latency x_2_ (ms)	7.527	(−1.624, 16.677)	0.101
Initial Latency x_3_ (ms)	−10.205	(−19.374, −1.036)	0.031 *
Initial Time (day)	−0.133	(−0.402, 0.136)	0.314
Initial grip strength (lbs.)	0.742	(0.567, 0.916)	<0.001 *
Pinch strength			
Initial Latency x_1_	(Ref)		
Initial Latency x_2_ (ms)	3.072	(−0.800, 6.944)	0.113
Initial Latency x_3_ (ms)	−3.110	(−7.172, 0.951)	0.125
Initial Time (day)	−0.069	(−0.182, 0.044)	0.218
Initial pinch strength (lbs.)	0.706	(0.445, 0.968)	<0.001 *
Box and block			
Initial Latency x_1_	(Ref)		
Initial Latency x_2_ (ms)	2.829	(−7.520, 13.178)	0.573
Initial Latency x_3_ (ms)	−10.204	(−20.254, −0.154)	0.047 *
Initial Time (day)	−0.138	(−0.442, 0.167)	0.355
Initial box and block (n)	0.733	(0.477, 0.990)	<0.001 *
9-hole pegboard			
Initial Latency x_1_	(Ref)		
Initial Latency x_2_ (ms)	−0.201	(−0.538, 0.136)	0.206
Initial Latency x_3_ (ms)	0.170	(−0.379, 0.720)	0.495
Initial Time (day)	0.002	(−0.012, 0.016)	0.743
Initial 9 peg (log_ms)	0.469	(0.188, 0.750)	0.005 *

K-MBI, Korean version of Modified Barthel Index; β, Regression coefficient; 95% CI, The lower and upper bounds of the confidence interval; * mean *p* < 0.05.

## Data Availability

The original contributions presented in this study are included in the article.

## Abbreviations

The following abbreviations are used in this manuscript:

MEP	Motor Evoked Potential
MRC	Medical Research Council
MMT	Manual Muscle Test
K-MBI	Korean version of the Modified Barthel Index
HFT	Hand Function Test
SSEP	Somatosensory Evoked Potential
